# Anal, Penile, and Oral High-Risk HPV Infections and HPV Seropositivity in HIV-Positive and HIV-Negative Men Who Have Sex with Men

**DOI:** 10.1371/journal.pone.0092208

**Published:** 2014-03-20

**Authors:** Vera M. van Rijn, Sofie H. Mooij, Madelief Mollers, Peter J. F. Snijders, Arjen G. C. L. Speksnijder, Audrey J. King, Henry J. C. de Vries, Arne van Eeden, Fiona R. M. van der Klis, Hester E. de Melker, Marianne A. B. van der Sande, Maarten F. Schim van der Loeff

**Affiliations:** 1 Centre for Infectious Disease Control, National Institute for Public Health and the Environment, Bilthoven, the Netherlands; 2 Cluster of Infectious Diseases, Public Health Service of Amsterdam, Amsterdam, the Netherlands; 3 Department of Pathology, VU University Medical Center, Amsterdam, the Netherlands; 4 Center for Infection and Immunity Amsterdam (CINIMA), Academic Medical Center, University of Amsterdam, Amsterdam, the Netherlands; 5 Department of Dermatology, Academic Medical Center, University of Amsterdam, Amsterdam, the Netherlands; 6 Department of Internal Medicine, Jan van Goyen Medical Center, Amsterdam, the Netherlands; 7 Julius Center for Health Sciences and Primary Care, University Medical Center Utrecht, Utrecht, the Netherlands; University of Nebraska – Lincoln, United States of America

## Abstract

The effects of single or multiple concordant HPV infections at various anatomical sites on type-specific HPV seropositivity are currently unknown. In this cross-sectional study we assessed whether high-risk HPV infections at various anatomical sites (i.e., anal canal, penile shaft, and oral cavity), as well as concordant infections at multiple anatomical sites, were associated with type-specific seropositivity in HIV-positive and HIV-negative MSM.

MSM aged ≥18 years were recruited in Amsterdam, the Netherlands (2010–2011). Baseline anal, penile, and oral samples were analyzed for HPV DNA and genotyped using a highly sensitive PCR and reverse line blot assay. Virus-like particle (VLP) based multiplex immunoassay was used to asses HPV-specific serum antibodies against L1 VLPs. The associations between HPV infections and type-specific seropositivity of seven high-risk HPV types (7-hrHPV: types 16, 18, 31, 33, 45, 52, 58) were estimated using logistic regression analyses with generalized estimating equations. We found that 86% of 306 HIV-positive MSM and 62% of 441 HIV-negative MSM were seropositive for at least one 7-hrHPV type. 69% of HIV-positive and 41% of HIV-negative MSM were infected with at least one 7-hrHPV type at the anus, penis, or oral cavity. In multivariable analyses, 7-hrHPV seropositivity was associated with type-specific anal (and not penile) 7-hrHPV infection, and did not significantly increase with a higher number of infected anatomical sites. Oral 7-hrHPV infection showed a positive, albeit non-significant, association with seropositivity. In conclusion, seropositivity among MSM appears to be largely associated with anal HPV infection, irrespective of additionally infected anatomical sites.

## Introduction

Human papillomavirus (HPV) infection is one of the most common sexually transmitted infections worldwide [1]. Persistent infection with high-risk HPV types is a leading cause of anogenital cancers and of a subset of oropharyngeal cancers [2]. A high prevalence of anal, penile, and oral HPV infections has been observed among men who have sex with men (MSM) [3]–[6] with an even higher prevalence among HIV-positive MSM [5]–[10].

In the majority of individuals an HPV infection is cleared within 4–20 months [11], [12]. Individuals naturally infected with HPV do not always develop antibody responses over time [13], [14]. If seroconversion does occur, antibodies may persist for many years [15]. Seropositivity is thought to be associated with persistent HPV infection, HPV viral load, and anatomical site of infection [13], [16]–[22].

Previous studies observed that HPV seropositivity was positively associated with type-specific anal HPV infection rather than with genital (penile) HPV infection [20]–[22]. Also, seropositivity was higher among HIV-positive than HIV-negative MSM [23]. However, studies on the association between high-risk HPV infections at various anatomical sites and type-specific HPV seropositivity in MSM with and without HIV infection are scarce. Moreover, to the best of our knowledge no study has investigated the associations of concordant infections at multiple anatomical sites with HPV seropositivity. Since HPV antibodies are generally regarded as a marker of lifetime HPV exposure, more insight into antibody responses will assist in the interpretation of sero-epidemiological studies and in targeting HPV prevention strategies.

In this cross-sectional study we assessed whether high-risk HPV infections at various anatomical sites (i.e., anal canal, penile shaft, and oral cavity), as well as concordant infections at multiple anatomical sites, are associated with type-specific seropositivity in HIV-positive and HIV-negative MSM.

## Materials and Methods

### Ethics statement

The Medical Ethics Committee of the Academic Medical Center (AMC) in Amsterdam approved the study. Written informed consent was obtained from all participants prior to enrollment.

### Study population

This analysis is based on baseline data of the HIV & HPV in MSM (H2M) study, a prospective cohort study which aims to compare the prevalence, incidence, and clearance of anal, penile, and oral HPV infections in HIV-positive and HIV-negative MSM.

Details of the H2M study and study population have been described previously [5]. In brief, HIV-positive and HIV-negative MSM were recruited from three sites in Amsterdam, the Netherlands: the Amsterdam Cohort Study among MSM [24], an STI clinic (both at the Public Health Service of Amsterdam), and an outpatient infectious disease clinic (Jan van Goyen Medical Center), between July 2010 and July 2011. Eligibility criteria included an age of 18 years or older, being male, having had sex with men, and competence in Dutch or English. In this cross-sectional analysis of baseline data, only MSM with available questionnaire data and complete baseline results (anal, penile, oral, and serum sample results) were included.

### Sample and data collection

A self-administered questionnaire was used to assess socio-demographic characteristics, general health-related issues (e.g., age, circumcision status, and smoking behavior), and details of lifetime and recent sexual behavior. Self-swabs of the anal canal and penile shaft, and oral swish-and-gargle samples were obtained for HPV DNA detection. Participants self-sampled the anus by inserting a dry swab (regular flocked swab with 1 ml UTM medium, Copan, Brescia, Italy) 3 cm into the anal canal and rotating it for 5–10 seconds. Penile self-samples were obtained by rubbing a dry swab firmly over the penile shaft, including the foreskin, for 20 seconds. Each dry swab was immediately put into the preservative UTM medium. For oral samples, participants were instructed to rinse the oral cavity and gargle for 30 seconds, using 10–15 ml Scope mouthwash (Procter & Gamble, Toronto, Ontario). A venous blood sample was collected for serum antibody testing. HIV-related data (CD4 cell count, HIV viral load, and use of combination antiretroviral therapy (cART)) were obtained from the Dutch HIV Monitoring Foundation’s national HIV patient database.

### HPV DNA detection and genotyping

Anal, penile, and oral samples were stored at –20°Celsius. Preceding storage, oral samples passed a washing and concentration step as described by Mooij et al. [5]. DNA extraction was conducted using MagNA Pure LC Total Nucleic Acid Isolation Kit (Roche, Mannheim, Germany). 200 μl UTM or oral medium was used as input; DNA was eluted in 100 μl buffer. Broad-spectrum HPV DNA amplification was conducted on 10 μl DNA extract using the highly sensitive SPF_10_-PCR (version 1) [25], which amplifies a 65 base pair open reading frame of the L1 region of the HPV genome. DNA enzyme-linked immunoassay (HPV-DEIA, Labo Biomedical Products, Rijswijk, the Netherlands) was used to detect amplified PCR products. PCR-DEIA-negative samples were considered HPV-negative; PCR-DEIA-positive samples were considered HPV-positive. HPV-positive amplicons were subsequently analyzed in a reverse line blot assay (HPV-LiPA_25_, Labo Biomedical Products, Rijswijk, the Netherlands). LiPA_25_ allows simultaneous detection of 25 specific mucosal HPV genotypes.

### HPV serum antibody testing

Serum samples were stored at –20°C and transported to the National Institute for Public Health and the Environment (RIVM, Bilthoven, the Netherlands), where samples were stored at –80°C until processing. Virus-like particle (VLP) based multiplex immunoassay was used to asses HPV-specific serum antibodies against L1 VLPs of genotypes 16, 18, 31, 33, 45, 52, 58, as previously described [26]. Serum samples were assumed to be HPV-seropositive at cut-offs previously determined using a 99% prediction interval method based on children aged 1–10 years [27]. Cut-off values were determined at ≥9, ≥13, ≥27, ≥11, ≥19, ≥14, ≥31 Luminex Units/milliliter (LU/ml) for HPV 16, 18, 31, 33, 45, 52 and 58, respectively.

### Nomenclature

We here report data regarding the seven high-risk HPV types which could be detected both by serology and LiPA_25,_ i.e., types 16, 18, 31, 33, 45, 52, and 58. Samples positive for one or more of these seven HPV types were designated 7-hrHPV-positive; samples negative for all of these seven high-risk types were categorized as 7-hrHPV-negative.

7-hrHPV infections were designated ‘concordant infections at multiple anatomical sites’ when HPV DNA of *the same* HPV type was detected at 2 or 3 sites within one individual: i.e., anal and penile; anal and oral; penile and oral; or anal, penile, and oral HPV infections.

### Statistical analyses at participant level

Characteristics of HIV-positive and HIV-negative MSM were compared using Chi-square tests for categorical data and Wilcoxon-Mann-Whitney rank-sum tests for continuous data. 95% confidence intervals (95% CI) were calculated for all 7-hrHPV infection prevalence and seroprevalence estimates. Since previous studies observed that risk factors for HPV infections and HPV seropositivity differed between HIV-positive and HIV-negative participants [5], [6], [9], [28]–[31], further analyses were stratified by HIV status. All statistical tests were two-sided. Differences were considered statistically significant at *P*<0.05.

### Statistical analyses at HPV-type infection level

Univariable and multivariable logistic regression analyses with generalized estimating equations (GEE) were used to study associations between 7-hrHPV infections at various anatomical sites and type-specific 7-hrHPV seropositivity, and to estimate associations between concordant 7-hrHPV infections at multiple anatomical sites and type-specific 7-hrHPV seropositivity.

GEE models are capable of estimating the exposure effect on multiple measurements in one individual, while taking into account the correlation between these multiple measurements [32]. In our analyses, these multiple measurements were HPV infections of the seven HPV types. An exchangeable correlation structure was used, since the correlation between pairs of 7-hrHPV types was assumed to be similar.

The following variables were identified in previous analyses [5], [6], [31] and were included in the multivariable regression analyses a priori: age, smoking, poppers use (alkyl nitrites) in the preceding 6 months, circumcision status, lifetime number of male sex partners, unprotected anal intercourse in the preceding 6 months, receptive anal intercourse in the preceding 6 months, active oral-anal contact in the preceding 6 months, and additionally for HIV-positive participants: most recent HIV viral load and CD4 cell count.

In the analyses assessing the associations between 7-hrHPV infections at various anatomical sites and type-specific seropositivity, we included baseline anal, penile, and oral 7-hrHPV infections within the same models, to be able to estimate the independent effect of 7-hrHPV infection at each anatomical site. In the analyses assessing the associations between concordant 7-hrHPV infections at multiple anatomical sites and type-specific seropositivity, we included a variable containing the number of concordant infections (i.e., no infection, infection at one anatomical site (single infection), or concordant infections at multiple anatomical sites) in the models.

Analyses were stratified by HIV status. In case of >40 missing values per variable in overall analyses, and >20 missing values in stratified analyses, an extra category for missing values was introduced to avoid loss of observations. All statistical analyses were performed using SAS software package version 9.3 (SAS Institute Inc., Cary, NC, USA).

## Results

### Characteristics of study population

The H2M study included 795 MSM; 747 of these men (94%) with available questionnaire data and anal, penile, oral, and serological sample results were included in this analysis. The 48 excluded participants did not differ from the included population in terms of age and HIV status. Out of the 747 MSM, 306 (41%) were HIV-positive and 441 (59%) were HIV-negative at enrollment. Baseline characteristics stratified by HIV status are shown in Table 1. The median age of HIV-positive MSM was 46 years compared to 38 years for HIV-negative MSM (*P*<0.001). HIV-positive MSM and HIV-negative MSM differed significantly in various characteristics: HIV-positive MSM were more likely to have been born outside of the Netherlands, to be smokers of tobacco, and had used poppers more often in the preceding six months compared to HIV-negative MSM. In addition, HIV-positive MSM reported more sexual risk behavior, including a higher lifetime number of male sex partners. At enrollment, most HIV-positive MSM were receiving cART (87%; 228/261), had a CD4 cell count above 350 cells/mm^3^ (85%; 208/246), and had an undetectable HIV viral load (78%; 199/254).

**Table 1:** Baseline characteristics of 747 MSM participating in the H2M study, overall and stratified by HIV status (Amsterdam, 2010–2011).^a^

		Total population		HIV-positive MSM		HIV-negative MSM		*P*-value b
		(N = 747)		(N = 306)		(N = 441)		
**Demographics**								
**Median age in years (IQR)**		40.1	(34.8–47.5)	45.6	(39.4–52.5)	37.6	(33.6–42.2)	**<0.001** c
**Age at enrollment (years) by category**								**<0.001**
	<35	189	(25.3)	41	(13.4)	148	(33.6)	
	35–44	331	(44.3)	103	(33.7)	228	(51.7)	
	≥45	227	(30.4)	162	(52.9)	65	(14.7)	
**Country of birth**								**0.048**
	Netherlands	601	(81.1)	237	(77.7)	364	(83.5)	
	Other	140	(18.9)	68	(22.3)	72	(16.5)	
**Health**								
**Tobacco smoking** d								**0.007**
	Never	249	(36.8)	78	(29.6)	171	(41.4)	
	Ever/in the past	177	(26.1)	74	(28.0)	103	(24.9)	
	Currently	251	(37.1)	112	(42.4)	139	(33.7)	
**Poppers use in the preceding 6 months**								**<0.001**
	No	358	(55.7)	120	(41.4)	238	(67.4)	
	Yes	285	(44.3)	170	(58.6)	115	(32.6)	
**Being circumcised**								0.151
	No	597	(81.2)	237	(78.7)	360	(83.0)	
	Yes	138	(18.8)	64	(21.3)	74	(17.1)	
**Sexual Behavior**								
**Median lifetime number of male sex partners (IQR)**		200	(60–600)	300	(100–1000)	100	(50–400)	**<0.001** c
**Lifetime number of male sex partners by category**								**<0.001**
	≤100	283	(40.7)	76	(26.9)	207	(50.2)	
	101–500	237	(34.1)	109	(38.5)	128	(31.1)	
	≥501	175	(25.2)	98	(34.6)	77	(18.7)	
**Unprotected anal intercourse in the preceding 6 months**								**<0.001**
	No anal intercourse	121	(16.5)	51	(16.9)	70	(16.1)	
	Anal intercourse, always protected	186	(25.3)	65	(21.6)	121	(27.9)	
	Anal intercourse, sometimes protected	305	(41.5)	150	(49.8)	155	(35.7)	
	Anal intercourse, never protected	123	(16.7)	35	(11.6)	88	(20.3)	
**Receptive anal intercourse in the preceding 6 months**								0.077
	No	248	(33.6)	90	(29.9)	158	(36.2)	
	Yes	490	(66.4)	211	(70.1)	279	(63.8)	
**Active oral-anal contact in the preceding 6 months**								0.938
	No	259	(35.4)	107	(35.6)	152	(35.3)	
	Yes	473	(64.6)	194	(64.5)	279	(64.7)	
**HIV**								
**Use of cART at enrollment**								
	No			33	(12.6)			
	Yes			228	(87.4)			
**HIV viral load at enrollment (copies/mL) by category**								
	<50			199	(78.4)			
	≥50			55	(21.7)			
**CD4 cell count in cells/mm^3^ by category**								
	≤350			38	(15.5)			
	>350			208	(84.6)			

Abbreviations: MSM = men who have sex with men; H2M = HIV & HPV in MSM; IQR = interquartile range; cART = combination antiretroviral therapy.

Significant results (*P*<0.05) are represented in bold font.

a Numbers do not always add up to the total because of missing values.

b Based on Chi-square test (except when stated otherwise).

c Based on rank-sum test.

d Smoking status of participants who were not current smokers but provided no information on past smoking behavior was regarded as missing.

### Participant level analysis: 7-hrHPV prevalence

7-hrHPV seroprevalence for at least one of the seven HPV types was higher in HIV-positive MSM compared to HIV-negative MSM (86% versus 62%; *P*<0.001). In both HIV-positive and HIV-negative MSM, anal 7-hrHPV infections were the most prevalent (43%), followed by penile (16%) and oral (10%) infections. Prevalence of anal, penile, and oral infections was significantly higher in HIV-positive MSM compared to HIV-negative MSM (*P*<0.001 for each comparison) (Table 2).

**Table 2:** Prevalence of 7-hrHPV infections at the anal canal, penile shaft, and oral cavity, and HPV seroprevalence in 747 MSM participating in the H2M study, overall and stratified by HIV status (Amsterdam, 2010–2011).

		Total population		HIV-positive MSM		HIV-negative MSM		*P*-value a
		(N = 747)		(N = 306)		(N = 441)		
		**n**	**(%)**	**n**	**(%)**	**n**	**(%)**	
**7-hrHPV seroprevalence** b		535	(71.6)	264	(86.3)	271	(61.5)	**<0.001**
**Anal, penile, or oral 7-hrHPV prevalence** c		391	(52.3)	212	(69.3)	179	(40.6)	**<0.001**
**7-hrHPV prevalence per anatomical site** c								
	Anal HPV	322	(43.1)	174	(56.9)	148	(33.6)	**<0.001**
	Penile HPV	120	(16.1)	71	(23.2)	49	(11.1)	**<0.001**
	Oral HPV	72	(9.6)	53	(17.3)	19	(4.3)	**<0.001**

Abbreviations: 7-hrHPV = human papillomavirus types 16, 18, 31, 33, 45, 52, 58; MSM = men who have sex with men; H2M = HIV & HPV in MSM.

Significant results (*P*<0.05) are represented in bold font.

a Based on Chi-square test.

b Participants seropositive for at least one of seven high-risk HPV types (16, 18, 31, 33, 45, 52 and/or 58).

c Participants positive for at least one of seven high-risk HPV types (16, 18, 31, 33, 45, 52 and/or 58).

### HPV-type infection level analysis: relative distribution of 7-hrHPV infections at anatomical sites

**Figure 1:**
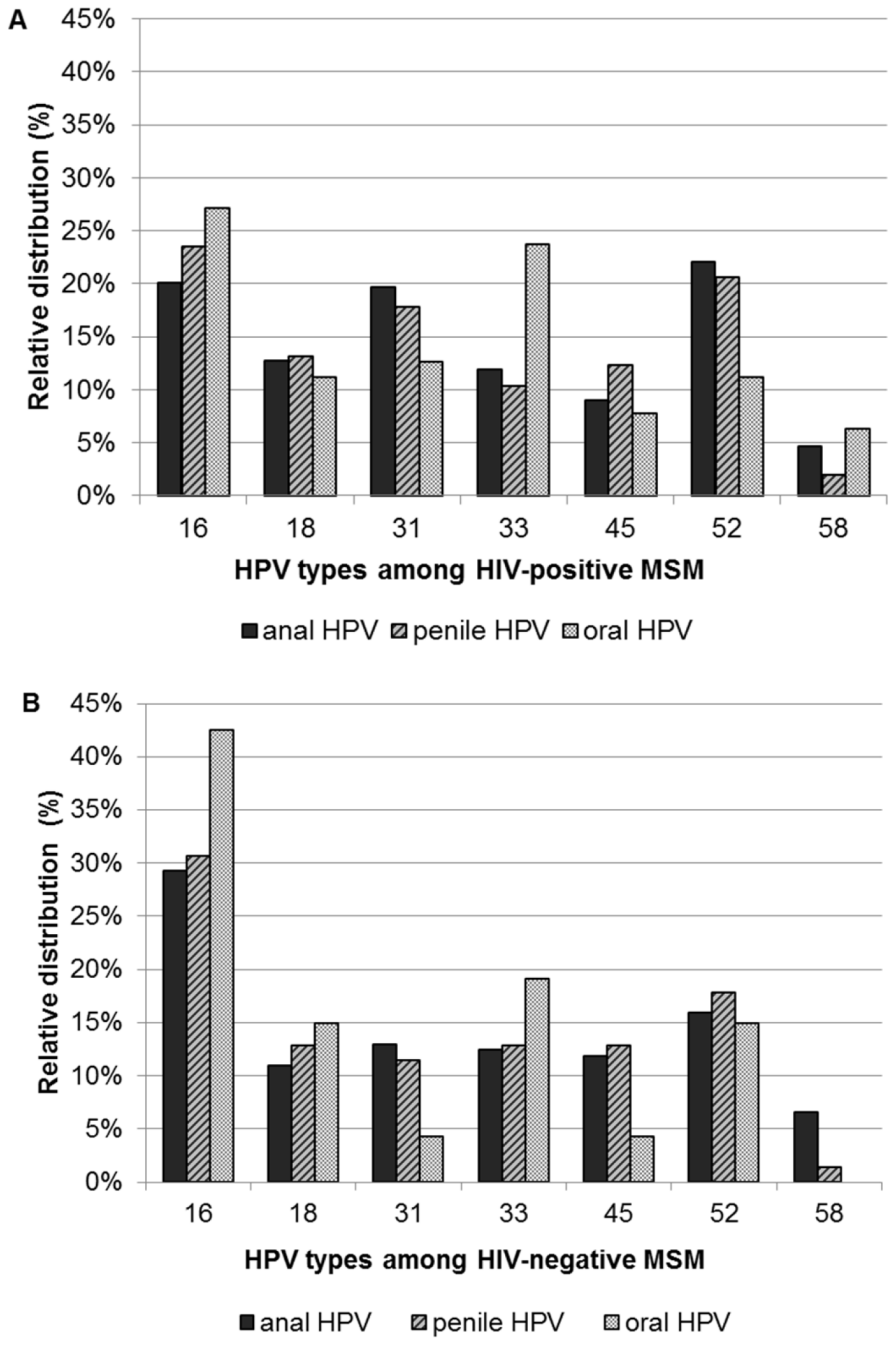
Relative type distribution among anal, penile, and oral HPV infections. Relative type distribution among anal, penile, and oral 7-hrHPV infections in HIV-positive (Figure 1A) and HIV-negative (Figure 1B) MSM participating in the H2M study (Amsterdam, 2010–2011). Please note that these are *relative* percentages, indicating e.g. which percentage of anal infections was HPV-16, which percentage was HPV-18 etc. The percentages anal infections for all 7 types add up to 100%. The same applies to penile and oral infections. It can be seen that among HIV-negative MSM the relative contribution of HPV-16 is larger for all three anatomical sites, compared to the situation in HIV-positive MSM. Abbreviations: 7-hrHPV = human papillomavirus types 16, 18, 31, 33, 45, 52, 58; MSM = men who have sex with men; H2M = HIV & HPV in MSM.

The relative type distribution among anal, penile, and oral 7-hrHPV infections, stratified by HIV status, is shown in Figure 1. In both HIV-positive and HIV-negative MSM, the distribution of oral 7-hrHPV types differed from that found in anal and penile samples. For example, in HIV-positive MSM, HPV-33 was detected relatively more often in oral (24%) samples than in anal (12%) and penile (10%) samples. In HIV-negative MSM, especially HPV-16 was more frequently detected in oral (43%) samples compared to anal (29%) and penile (31%) samples. Among HIV-negative MSM, a larger proportion of all infections was caused by HPV-16 than among HIV-positive MSM (43% versus 27%, respectively, *P* = 0.078).

**Figure 2:**
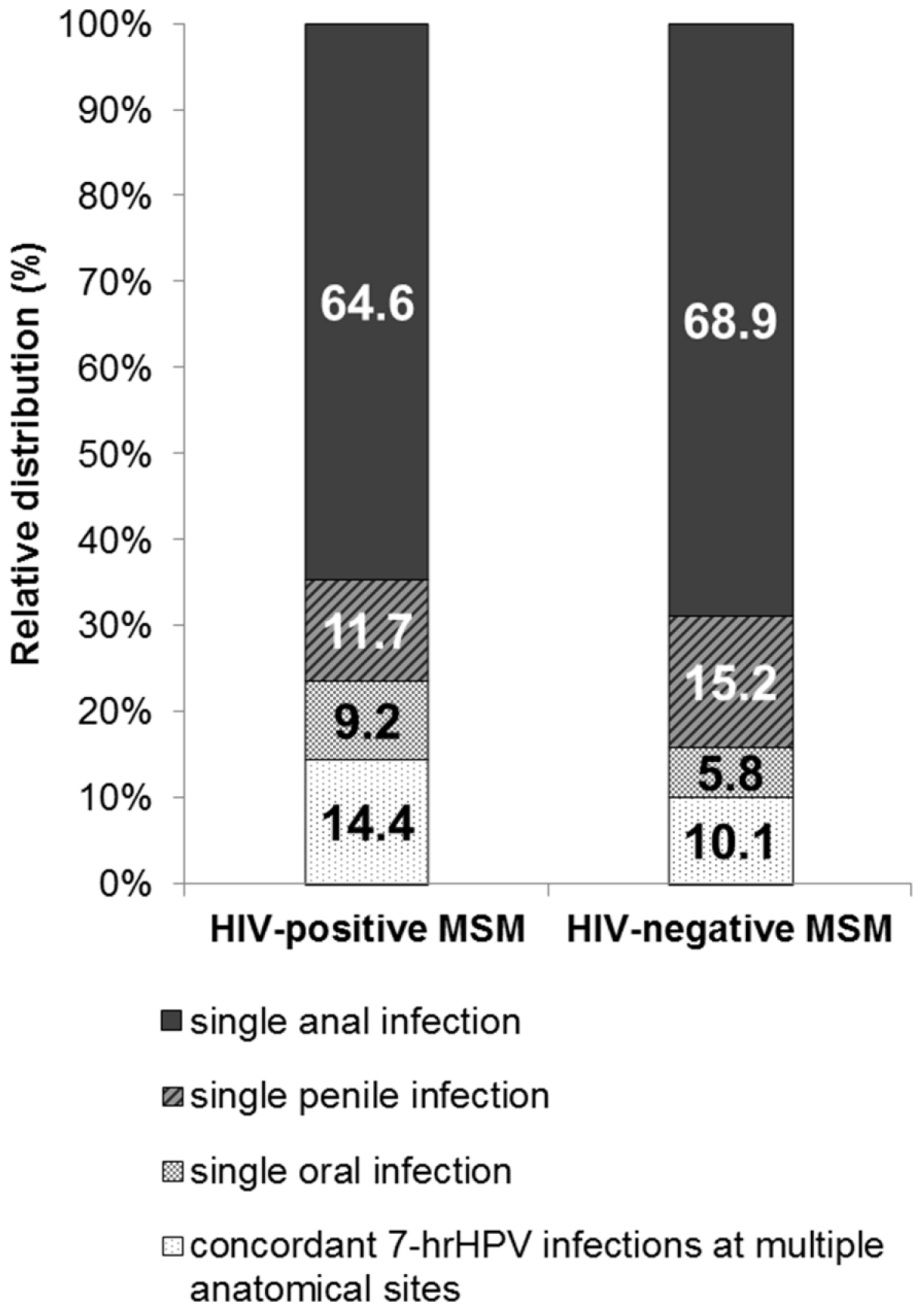
Relative distribution of HPV infections at one or multiple anatomical sites. Relative distribution of 7-hrHPV infections at only one anatomical site (single infection) and concordant infections at multiple anatomical sites in HIV-positive and HIV-negative MSM participating in the H2M study (Amsterdam, 2010–2011). Please note that, per HPV type, each participant could have no infection, infection at one anatomical site, or infections at multiple anatomical sites. Abbreviations: 7-hrHPV = human papillomavirus types 16, 18, 31, 33, 45, 52, 58; MSM = men who have sex with men; H2M = HIV & HPV in MSM.

The relative distribution of 7-hrHPV infection at one anatomical site (single infection) versus concordant infections at multiple anatomical sites, stratified by HIV status, is shown in Figure 2. In both HIV-positive and HIV-negative MSM 7-hrHPV infections were more often detected at one anatomical site than at multiple sites. Concordant 7-hrHPV infections detected at multiple anatomical sites comprised 14% and 10% of the 7-hrHPV infections in HIV-positive and HIV-negative MSM, respectively *(P* = 0.101).

### Risk factors for concordant 7-hrHPV infections at multiple anatomical sites

In univariable and multivariable analyses, neither HIV status, nor any of the a priori risk factors was significantly associated with concordant 7-hrHPV infections at multiple anatomical sites compared to infection at one anatomical site (single infection). In addition, analyses stratified by HIV status found that none of the a priori risk factors were associated with concordant 7-hrHPV infections at multiple anatomical sites (data not shown). In HIV-negative MSM, lifetime number of male sex partners reached borderline significance in univariable (odds ratio (OR) 5.0; 95% CI 1.0–25.4 for ≥501 partners compared to ≤100, overall *P* = 0.066) and multivariable analysis (adjusted odds ratio (aOR) 5.2; 95% CI 0.8–32.6 for ≥501 partners compared to ≤100, overall *P* = 0.081).

### Associations between 7-hrHPV infections at various anatomical sites and type-specific seropositivity

Univariable and multivariable analyses of associations between anal, penile, and oral 7-hrHPV infection and type-specific seropositivity are shown in Table 3. Anal 7-hrHPV infection was independently associated with seropositivity in overall multivariable analysis (aOR 1.8; 95% CI 1.5–2.2). Penile 7-hrHPV infection showed a small positive association, but was not significantly associated with seropositivity in either univariable or multivariable analyses. Oral 7-hrHPV infection showed increased odds in both univariable and multivariable analyses, although the association was only significant in univariable analysis (OR 1.6; 95% CI.1.0–2.5).

**Table 3:** Univariable and multivariable analyses of associations between anal, penile, and oral 7-hrHPV infections and type-specific seropositivity in 747 MSM participating in the H2M study (Amsterdam, 2010–2011).

			Associations with seropositivity in total population (N = 747)			
		Number of infections	OR (95% CI)	*P*-value	aOR (95% CI)	*P*-value
**7-hrHPV infections**		**798**				
	Anal	546	1.91 (1.61–2.27)	<0.001	1.79 (1.49–2.16)	<0.001
	Penile	168	1.21 (0.92–1.58)	0.178	1.12 (0.84–1.48)	0.440
	Oral	84	1.60 (1.04–2.47)	0.032	1.41 (0.89–2.22)	0.143
			**Associations with seropositivity in HIV-positive MSM (N = 306)**			
**7-hrHPV infections**		**514**				
	Anal	345	1.57 (1.27–1.96)	<0.001	1.64 (1.30–2.08)	<0.001
	Penile	106	1.25 (0.89–1.75)	0.203	1.22 (0.85–1.74)	0.285
	Oral	63	1.53 (0.90–2.58)	0.114	1.57 (0.89–2.77)	0.121
			**Associations with seropositivity in HIV-negative MSM (N = 441)**			
**7-hrHPV infections**		**284**				
	Anal	201	2.36 (1.77–3.14)	<0.001	2.24 (1.65–3.03)	<0.001
	Penile	62	1.06 (0.65–1.75)	0.811	1.00 (0.60–1.64)	0.984
	Oral	21	1.39 (0.64–3.01)	0.408	1.06 (0.46–2.44)	0.893

The following variables were included in the multivariable models: age, smoking, poppers use, circumcision status, lifetime number of male sex partners, recent unprotected anal intercourse, recent receptive anal intercourse, recent active oral-anal contact, baseline anal, penile, and oral 7-hrHPV infections (and additionally for HIV-positive MSM: HIV viral load and CD4 cell count).

Abbreviations: 7-hrHPV = human papillomavirus types 16, 18, 31, 33, 45, 52, 58; MSM = men who have sex with men; H2M = HIV & HPV in MSM; OR = odds ratio; aOR = adjusted odds ratio; 95% CI = 95% confidence interval.

Significant results (*P*<0.05) are represented in bold font.

In multivariable analyses stratified by HIV status, anal 7-hrHPV infection was significantly associated with seropositivity in both HIV-positive (aOR 1.6; 95% CI 1.3–2.1) and HIV-negative MSM (aOR 2.2; 95% CI 1.7–3.0). Neither penile, nor oral 7-hrHPV infections were significantly associated with seropositivity in either HIV-positive or HIV-negative MSM, although in HIV-positive MSM the strength of the association of oral 7-hrHPV infection (aOR 1.6; 95% CI 0.9–2.8) was comparable with that of anal infection.

### Associations between concordant 7-hrHPV infections at multiple anatomical sites and type-specific seropositivity

Compared to having no infection, we found that in overall univariable and multivariable analyses both 7-hrHPV infection at one anatomical site and concordant 7-hrHPV infections at multiple anatomical sites were significantly associated with type-specific seropositivity (Table 4). In stratified multivariable analyses, the association between 7-hrHPV infection at one anatomical site and seropositivity was significant in both HIV-positive and HIV-negative MSM (aOR 1.5; 95% CI 1.2–1.9 and aOR 1.9; 95% CI 1.4–2.6, respectively). Concordant 7-hrHPV infections at multiple anatomical sites were significantly associated with seropositivity in HIV-positive MSM (aOR 2.2; 95% CI 1.5–3.4) and reached borderline significance in HIV-negative MSM (aOR 1.8; 95% CI 1.0–3.4).

**Table 4:** Univariable and multivariable analyses of associations between concordant 7-hrHPV infections at one or multiple anatomical sites and type-specific seropositivity in 747 MSM participating in the H2M study (Amsterdam, 2010–2011).

			Associations with seropositivity in total population (N = 747)					
		Number of observations	OR (95% CI)	*P*-value		aOR (95% CI)		*P*-value
**7-hrHPV infections at one or multiple anatomical sites**								
	No infection	4528	1.0			1.0		
	Infection at one anatomical site	611	1.77 (1.50–2.09)	**<0.001**		1.63 (1.37–1.94)		<0.001
	Infections at multiple anatomical sites	90	2.33 (1.66–3.26)	**<0.001**		2.00 (1.41–2.83)		<0.001
			**Associations with seropositivity in HIV-positive MSM (N = 306)**					
**7-hrHPV infections at one or multiple anatomical sites**								
No infection		1698	1.0			1.0		
Infection at one anatomical site		380	1.49 (1.22–1.84)	**<0.001**		1.52 (1.22–1.90)		<0.001
Infections at multiple anatomical sites		64	2.11 (1.40–3.19)	**<0.001**		2.23 (1.45–3.42)		<0.001
			**Associations with seropositivity in HIV-negative MSM (N = 441)**					
**7-hrHPV infections at one or multiple anatomical sites**								
No infection		2830	1.0				1.0	
Infection at one anatomical site		231	2.03(1.54–2.69)		**<0.001**		1.91 (1.43–2.56)	
Infections at multiple anatomical sites		26	2.40 (1.28–4.52)		**0.007**		1.80 (0.95–3.42)	

The following variables were included in the multivariable models: age, smoking, poppers use, circumcision status, lifetime number of male sex partners, recent unprotected anal intercourse, recent receptive anal intercourse, recent active oral-anal contact, concordant 7-hrHPV infections at one or multiple anatomical sites (and additionally for HIV-positive MSM: HIV viral load and CD4 cell count).

Abbreviations: 7-hrHPV = human papillomavirus types 16, 18, 31, 33, 45, 52, 58; MSM = men who have sex with men; H2M = HIV & HPV in MSM; OR = odds ratio; aOR = adjusted odds ratio; 95% CI = 95% confidence interval.

Significant results (*P*<0.05) are represented in bold font.

Concordant 7-hrHPV infections at multiple anatomical sites generally demonstrated a stronger association with type-specific seropositivity than infection at one anatomical site. However, a sub-analysis excluding observations with no infection showed that the odds of seropositivity were not significantly higher for concordant 7-hrHPV infections at multiple anatomical sites than for single infection in overall (*P* = 0.292) or stratified multivariable analyses (HIV-positive MSM: *P* = 0.829; HIV-negative MSM: *P* = 0.104).

## Discussion

In MSM, regardless of HIV status, 7-hrHPV seropositivity was associated with type-specific anal infection. No independent significant associations were observed between 7-hrHPV seropositivity and type-specific penile or oral infection, although oral infection in particular also showed a positive association with seropositivity. Seropositivity was associated with having a 7-hrHPV infection, but did not seem to increase with the number of infected anatomical sites.

The observations concerning anal and penile HPV infection and seropositivity are consistent with a recent study by Lu et al., regarding HPV-6 and HPV-16 infections and seropositivity in HIV-negative heterosexual men and MSM [21]. They observed that HPV-16 seropositivity was higher in MSM with an anal-only HPV-16 infection, than in MSM with a genital-only HPV-16 infection. It is hypothesized that infections in keratinized epithelium, which covers the glans and the shaft of the penis, may be less likely to elicit an immune response (and subsequently induce antibody production) than infections in mucosal epithelium, which covers the anal canal and oral cavity [21], [22], [33], [34]. Our findings regarding the anal and penile site of infection are in line with this hypothesis.

We could not demonstrate a significant independent association between oral HPV infection and seropositivity, although both the anal canal and oropharynx are lined by stratified squamous epithelium and may share certain biological characteristics. Possibly our study was underpowered to detect a significant independent association between oral HPV infection and seropositivity. A lack of concordance between oral HPV infection and type-specific serum antibodies among women has been described before [35]. Studies among men are scarce; one study suggested that tonsillar HPV infection may be associated with seropositivity in men, although this analysis was not performed on an HPV type-specific level [36].

Differences between anatomical sites may also be explained by other biological (e.g., regarding local immunity) or behavioral factors (e.g., degree of mucosal damage and HPV exposure upon sexual contact). The significant relationship between anal 7-hrHPV infection and seropositivity in this study may be caused by higher persistence and/or higher HPV viral load of anal HPV infection compared to penile or oral infection, as both persistence and higher HPV viral load may increase the probability of seroconversion [13], [16]–[19] as well as the chance of detecting an HPV infection at one time point. In longitudinal follow-up of this study population, we will further explore the temporal relationships between 7-hrHPV infections and seropositivity.

To the best of our knowledge, this study was the first to assess the association between the number of infected anatomical sites and HPV seropositivity in MSM. Unexpectedly, the odds for seropositivity were only slightly higher for concordant 7-hrHPV infections at multiple anatomical sites than for single infection at one anatomical site. We showed that anal 7-hrHPV infections were most frequently detected, and were independently associated with seropositivity, whereas penile or oral 7-hrHPV infections were not. Therefore, having an additional penile or oral 7-hrHPV infection was not likely to increase the chance of seropositivity.

We could not confirm that HIV status or any of the a priori risk factors identified from the literature were associated with having concordant infections at multiple anatomical sites rather than an infection at one anatomical site. The failure to determine any risk factors associated with the number of infected anatomical sites in our study may be due to limited power.

Compared to other HPV types, HPV-16, which causes the majority of all HPV-related cancers, was detected *relatively* more frequently in HIV-negative than in HIV-positive MSM (however, *absolute* HPV-16 prevalence was higher in HIV-positive than in HIV-negative MSM). This difference might be due to a combination of factors: HIV-related immunosuppression may lead to more persistent HPV infections, and less clearance of a wider spectrum of 7-hrHPV types in addition to HPV-16. Moreover, HIV-positive MSM may have a higher rate of acquisition of HPV types other than HPV-16 due to sexual risk behavior.

This study has two major strengths. The size of our study population was relatively large, and we were able to collect extensive information through detailed questionnaires concerning the socio-demographic and sexual behavior characteristics of our participants. Also, data pertaining to HPV infections at three anatomical sites and serology data were collected at the same time point for each participant, enabling us to look at various cross-sectional associations.

This study had some limitations. (1) We assessed presence of 7-hrHPV infections and concordant seropositivity at one time point only. A temporal relationship could therefore not be established. (2) HIV-positive and HIV-negative participants were recruited at separate study locations. We handled differences in baseline characteristics by adjusting for potential confounders. (3) This study may lack power due to limited number of oral and penile HPV infections, and our results should therefore be interpreted with caution. We were not able to estimate the impact of pairs of anatomical sites (concordant anal-penile, anal-oral, and penile-oral infections) on seropositivity. In addition, a difference in seropositivity between infections at one or multiple anatomical sites may have been difficult to assess, because of the very high seroprevalence among MSM.

In conclusion, our data suggest that in both HIV-positive and HIV-negative MSM, 7-hrHPV seropositivity is mainly associated with type-specific anal infection, and not penile or oral infection. In addition, seropositivity seems to be associated with having a 7-hrHPV infection, irrespective of the number of infected anatomical sites. As anal cancer and HPV-related oro-pharyngeal cancer seem to have been on the increase [37]-[39], insight into antibody responses by anatomical site may be helpful for interpreting sero-epidemiological studies and monitoring HPV prevention strategies, such as HPV vaccination.
